# Optimization of Substituted 6-Salicyl-4-Anilinoquinazoline Derivatives as Dual EGFR/HER2 Tyrosine Kinase Inhibitors

**DOI:** 10.1371/journal.pone.0069427

**Published:** 2013-08-01

**Authors:** Dong-Dong Li, Ya-Juan Qin, Jian Sun, Jing-Ran Li, Fei Fang, Qian-Ru Du, Yong Qian, Hai-Bin Gong, Hai-Liang Zhu

**Affiliations:** 1 State Key Laboratory of Pharmaceutical Biotechnology, Nanjing University, Nanjing, P. R. China; 2 Xuzhou Central Hospital, Xuzhou, P. R. China; University of Bologna & Italian Institute of Technology, Italy

## Abstract

4-Anilinoquinazolines as an important class of protein kinase inhibitor are widely investigated for epidermal growth factor receptor (EGFR) tyrosine kinase or epidermal growth factor receptor 2 (HER2) inhibition. A series of novel 6-salicyl-4-anilinoquinazoline derivatives **9–27** were prepared and evaluated for their EGFR/HER2 tyrosine kinase inhibitory activity as well as their antiproliferative properties on three variant cancer cell lines (A431, MCF-7, and A549). The bioassay results showed most of the designed compounds exhibited moderate to potent in vitro inhibitory activity in the enzymatic and cellular assays, of which compound **21** revealed the most potent dual EGFR/HER2 inhibitory activity, with IC_50_ values of 0.12 *µ*M and 0.096 *µ*M, respectively, comparable to the control compounds Erlotinib and Lapatinib. Furthermore, the kinase selectivity profile of **21** was accessed and demonstrated its good selectivity over the majority of the close kinase targets. Docking simulation was performed to position compound **21** into the EGFR/HER2 active site to determine the probable binding pose. These new findings along with molecular docking observations could provide an important basis for further development of compound **21** as a potent EGFR/HER2 dual kinase inhibitor.

## Introduction

**Figure 1:**
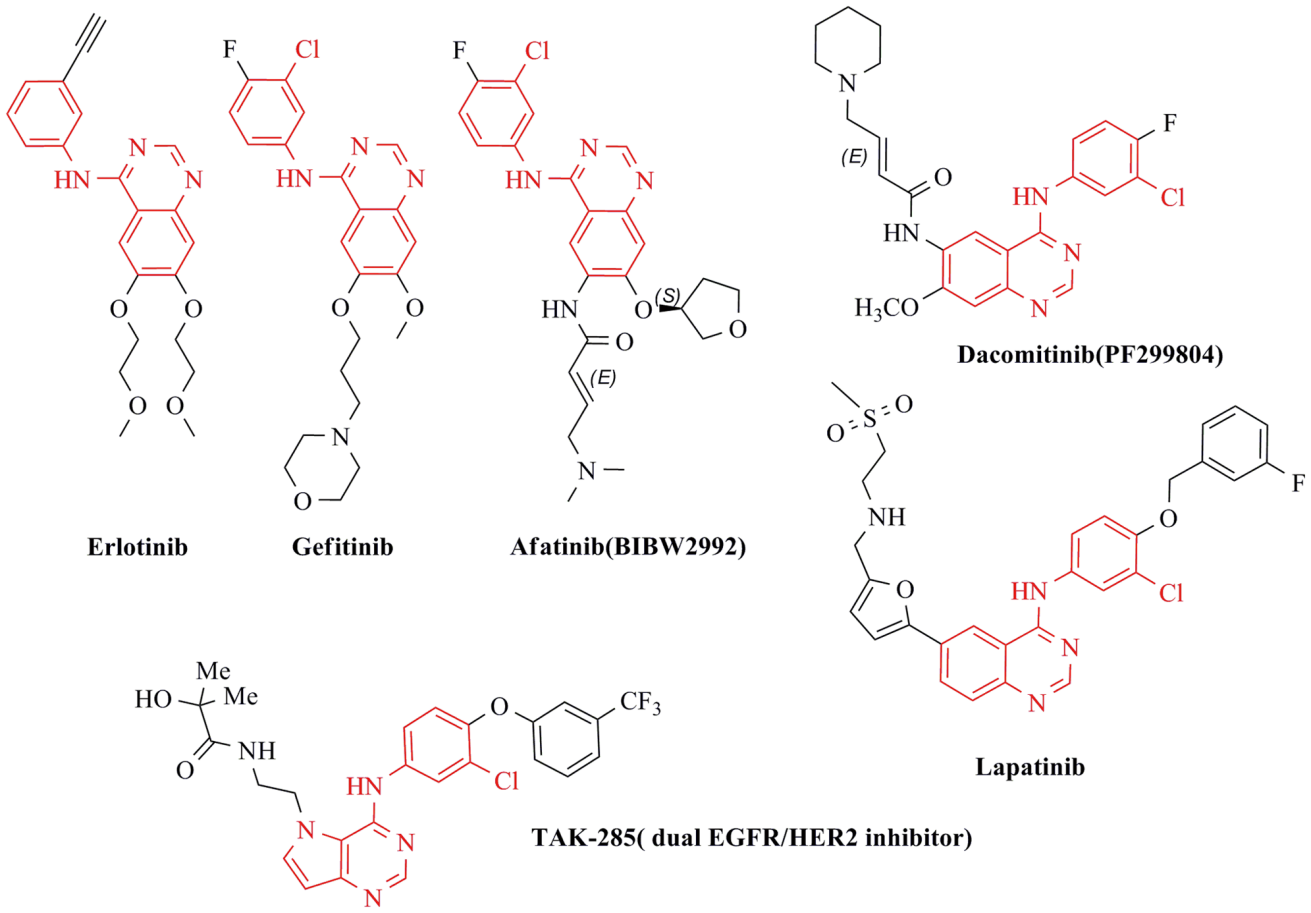
Structure of EGFR and/or HER2 small molecule inhibitors with 4-anilinoquinazoline scaffold.

The epidermal growth factor receptor (EGFR) family of receptor tyrosine kinase consists of human epidermal growth factor receptor (EGFR/ErbB-1), human epidermal growth factor receptor 2 (HER2/ErbB-2), human epidermal growth factor receptor 3 (Her3/Erb-3), and human epidermal growth factor receptor 4 (Her4/Erb-4), each of which was closely implicated in cell proliferation, survival, adhesion, migration and differentiation [1]. A number of researches demonstrated that overexpressions of EGFR and ErbB-2 has been observed in many cancer patients and correlate with a poor prognosis for several tumors including non-small-cell lung cancer (NSCLC), prostate, breast, stomach, colon, and ovarian cancers [2]–[6]. The blockade of EGFR and ErbB-2 has been clinically validated as an attractive approach for cancer therapy. As shown in Figure 1, the first-generation EGFR inhibitors Gefitinib (Iressa™) [4], [7] and Erlotinib (Tarceva™) [8] were approved for non-small-cell lung cancer (NSCLC) therapy, and Lapatinib as a dual reversible EGFR/HER2 inhibitor was approved for breast cancer therapy [9]. Two candidate drugs Afatinib [10], [11] and Dacomitinib [10], [12], which could bond with a unique cysteine797 residue located at the lip of the ATP binding cleft [13], as the most promising second generation EGFR kinase inhibitors, have been being evaluated in respective phase III study. Moreover, the pyrrolo [3,2-*d*]pyrimidine derivative TAK-285 [14], of which hinge binder was similar to the 4-anilinoquinazoline core, has also been developed as dual EGFR/HER2 inhibitor.

During our research for potent EGFR tyrosine kinase inhibitors, we previously reported a series of novel 4-anilinoquinazoline derivatives, mainly focusing on the modifications of the substituent at the 6- and 7-positions of the quinazoline ring [15], [16]. It is noteworthy that several known EGFR inhibitors (Figure 1), no matter ATP-competitive agents or ATP-noncompetitive/irreversible agents, need to share the basic structural motif of 4-anilinoquinazoline, which has been identified as a most versatile template for kinase inhibition [17]. Much of structural alterations of these compounds presented mostly exist at the 4-, 6-, and 7-positons of the basic quinazoline scaffold. Accordingly, a huge volume of researches on the synthesis, structure-activity relationships, and antiproliferative activities of the quinazoline-containing derivatives have been reported over the past ten years [18]–[21]. To our knowledge, the nitrogen at position 3 is important to EGFR inhibitory potency, but the nitrogen at position 1 is an even more important contributor [18]. Electron-donating substituents on the 6- and 7 positions could enhance the binding of N1 and N3, as do specifically substituted C4-anilines. In addition, structure activity relationships (SAR) in the quinazoline series have already demonstrated tolerance of bulky substituents at the 6- and 7-positions of the quinazoline ring [19].

**Figure 2:**
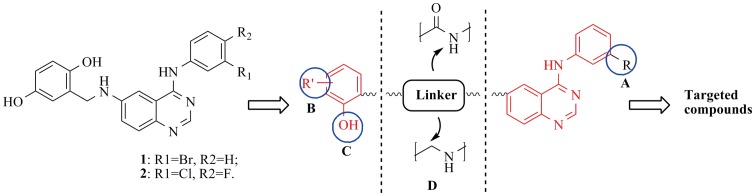
Optimization strategy for dual EGFR/HER2 inhibitors based on the structures of compound 1and 2. As shown in the picture, the main modifications would focus on the four parts of the basic 4-anilinoquinazoline scaffold (A, B, C, and D).

**Figure 3:**
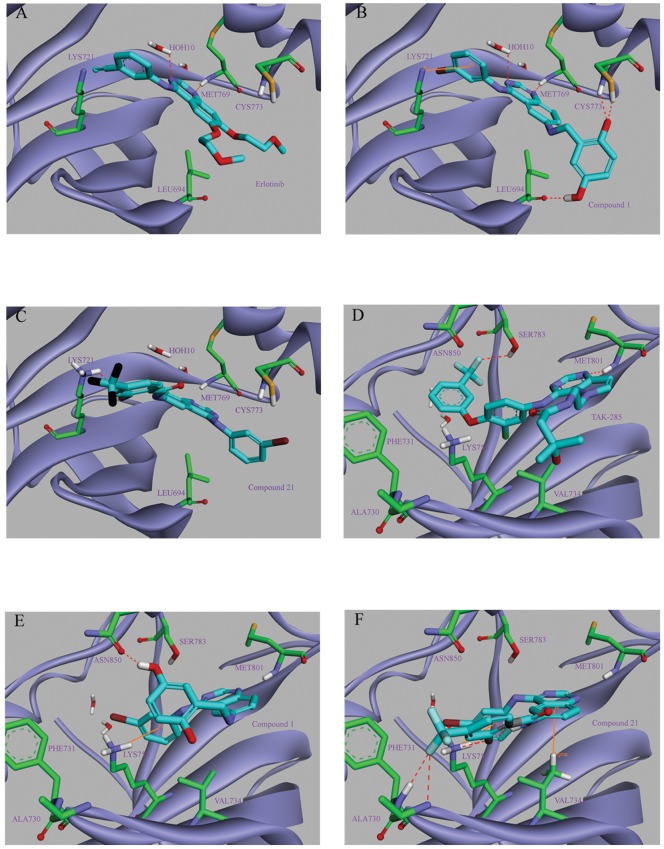
Docking model of the initial compound 1 and compound 21 bound to the active sites of the EGFR and HER2 proteins (PDB code: 1M17 and 3RCD, respectively). Inhibitor atoms colored as follows: C, Cyan; N, blue; O, red; key surrounding residues colored as follows: C, green; N, blue; O, red; S, yellow. (A) The binding mode of the control molecule Erlotinib in the binding site of raw EGFR protein crystal complex. It is noted that residue MET769 and HOH 10 could formed two hydrogen bonds with Erlotinib. (B) Compound **1** docked into the binding site of the EGFR protein. Its binding manner to the target protein seems very similar to the control Erlotinib. (C) The optimal docking pose of compound **21** into the ATP-binding pocket of the EGFR protein. (D) The binding conformation of the control molecule TAK-285 with the HER2 protein. Two residues MET801 and SER783 in the hinge binder play a key role in stabilizing ligand pose. (E) The optimal docking pose of compound **1** into the ATP-binding pocket of the HER2 protein. (F) The optimal docking pose of compound **21** into the ATP-binding pocket of the HER2 protein.

Among thousands of these 4-anilinoquinazolines, compound **1** and **2** empolying Lavendustin A subunit at the 6-position (Figure 2), reported by Albuschat *et al* [22] would be selected as the starting point for the development of dual EGFR/HER2 inhibitors in our study. Molecular docking studies based on EGFR and HER2 co-crystal structures (EGFR pdb code: 1M17, and HER2 pdb code: 3RCD) suggested that compound **1** would fit into the ATP-binding site of the HER2/EGFR protein (Figure 3A, 3B, 3D, and 3E). Our analysis of Figure 3A and Figure 3B suggested that in addition to the optimal binding pose similar to Erlotinib, introduction of o-hydroxyl group of Lavendustin A subunit could form two hydrogen bond interactions with CYS773 of the EGFR protein, which implied that this moiety was of importance for the binding potency. However, comparing Figure 3D with Figure 3E, it was seen that the binding mode of compound **1** to the HER2 protein was not similar with that of TAK-285, although the two hinge binders (pyrrolo [3,2-*d*]pyrimidine of TAK-285, and 4-anilinoquinazoline of compound **1**) were anchored at the same space of the HER2 protein. In order to develop novel 4-anilinoquinazoline derivatives as dual EGFR/HER2 inhibitors, we envisioned that four kinds of modifications (A, B, C, D) presented in Figure 2 based on the structure of compound **1** would generate novel potent inhibitors.

In this manuscript, we described the design and structure activity relationships (SAR) of a series of 6-salicyl-4-anilinoquinazoline analogs as dual EGFR/HER2 inhibitors and as antitumor agents. Molecular docking studies demonstrated key molecular interactions of the 6-salicyl-4-anilinoquinazoline scaffold with EGFR and HER2, which would provide additional clues for the development of novel EGFR/HER2-targeting therapeutic agents.

## Results and Discussion

### Molecular docking evaluation

**Figure 4:**
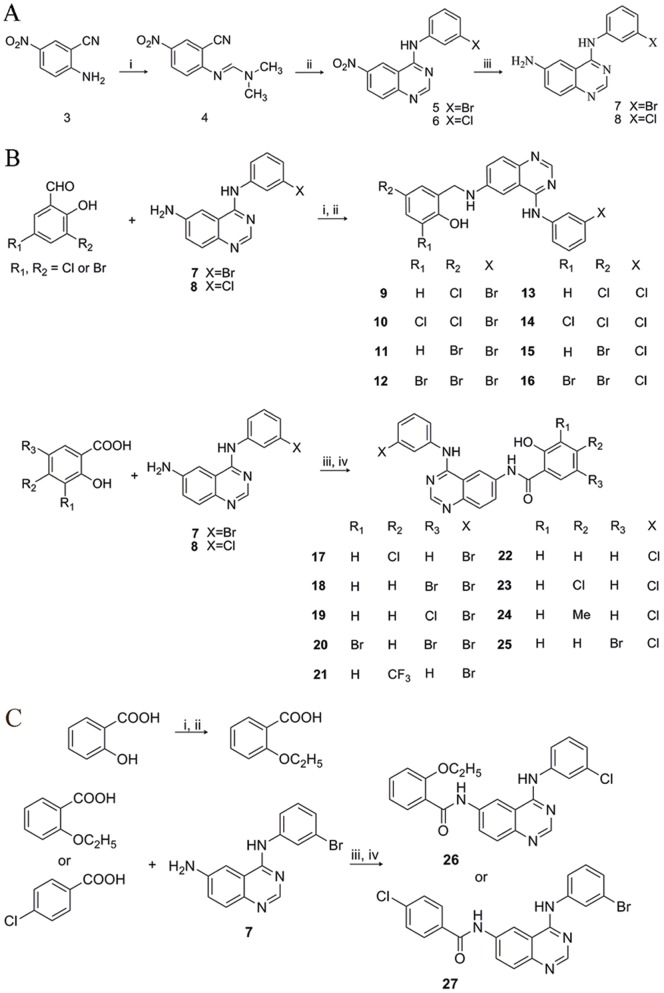
Synthesis of the designed compounds. (A) Synthesis of intermediates **3–8**. Reagents and conditions: (i) Dimethylformamide dimethyl acetal/70–75°C; (ii) ArNH_2_/AcOH/70–75°C; (iii) Fe/AcOH/EtOH/H_2_O/70–80°C. (B) Synthesis of compounds **9–25**. Reagents and conditions: (i) EtOH/40–60°C /3–4 h; (ii) EtOH/NaBH_4_/rt; (iii) SOCl_2_/70–80°C /reflux/4 h; (iv) EtOAC/K_2_CO_3_/ice-bath/overnight. (C) Synthesis of compounds **26** and **27**. Reagents and conditions: (i) C_2_H_5_I/K_2_CO_3_/DMF/80°C /6 h; (ii) NaOH/EtOH/reflux/4 h; (iii) SOCl_2_/70–80°C /reflux/4 h; (iv) EtOAC/K_2_CO_3_/ice-bath/overnight.

**Figure 5:**
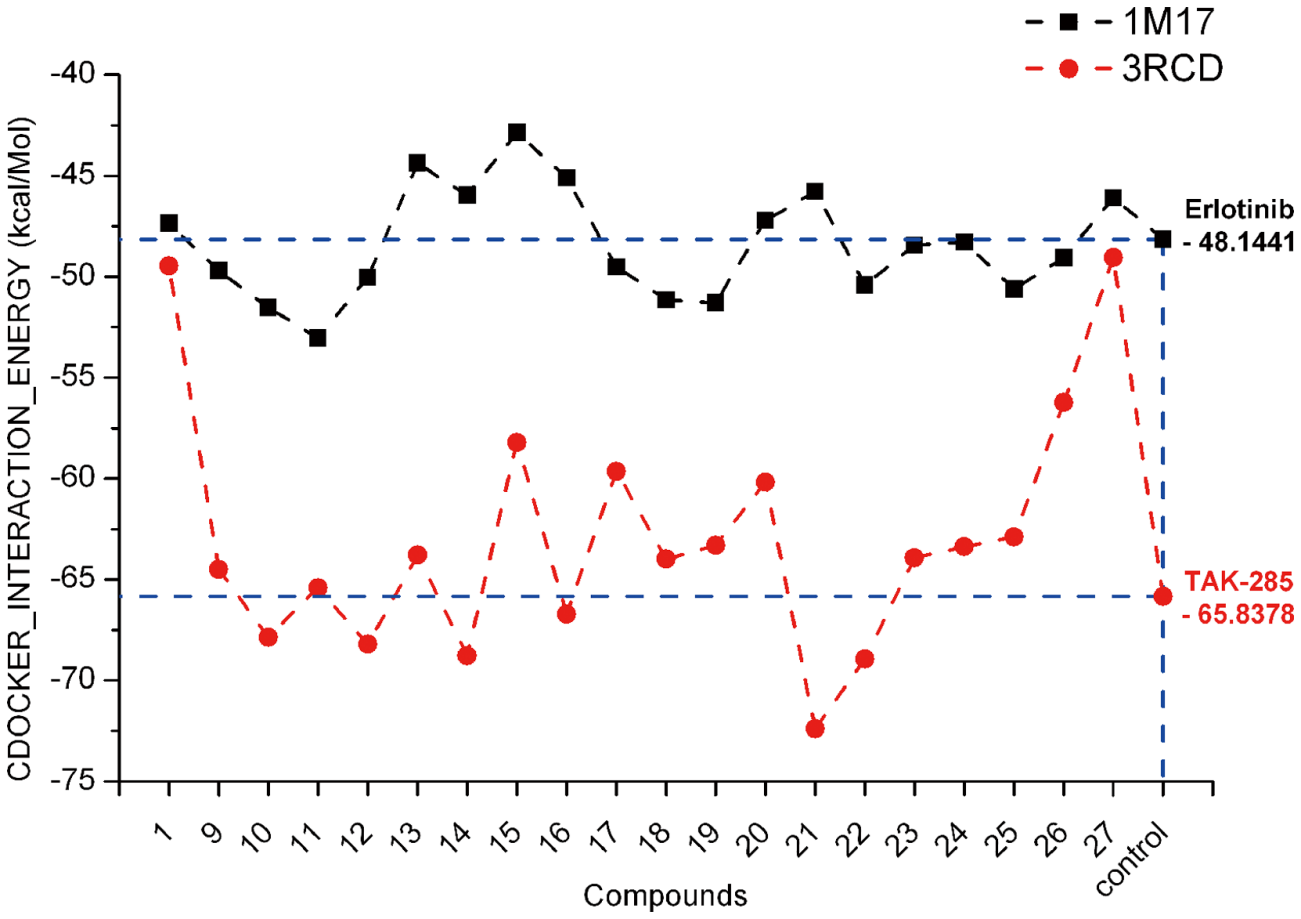
The CDOCKER_INTERACTION_ENERGY (kcal/mol) obtained from the docking study of compounds 9–27 by the CDOCKER protocol (Discovery Studio 3.5, Accelrys, Co. **Ltd).** These results were mainly derived from two molecular docking studies, in which one was performed between compounds **9–27**, Erlotinib and the EGFR protein (PDB code: 1M17), while the other one was performed between compounds **5–25**, TAK-285 and the HER2 protein (PDB code: 3RCD). This sort of energy was represented by a negative value, which indicates the value the lower, the outcome the better. Compared to Erlotinib and TAK-285, most of target compounds would display higher affinity for EGFR and HER2, respectively.

According to Figure 2 as well as considering chemical reagents available in our laboratory, 4-anilinoquinazoline compounds **7–25** (Figure 4) were designed, and virtually evaluated by the means of the CDOCKER protocol [23] in the Accelrys Discovery Studio 3.5 suite. Two groups of molecular dockings between these novel 4-anilinoquinazoline analogs and two protein-ligand complexes (as for EGFR, PDB code: 1M17; as for HER2, PDB code: 3RCD) retrieved from the RCSB Protein Data Bank were performed, and the obtained results were plotted as a line-scatter graph and presented in Figure 5, describing the CDOCKER_INTERACTION_ENERGY of two sets of molecular dockings. Compared with two positive drugs (Erlotinib and TAK-285), respectively, most of the designed molecules below the blue dash lines would possess lower interaction energy, demonstrating that they are likely to exhibit more potent against both of the two protein targets. The docking study performed on the 1M17 protein crystal structure was represented by a black solid line and as clearly seen in the picture, compounds **9–12**, **17–19**, and **22–26** showed lower interaction energy than Erlotinib that reached up to −48.1441 kcal/mol. On the other hand, the docking study based on the 3RCD protein crystal structure also indicated compounds **10**, **12**, **14**, **16**, **21**, **22** could exhibit more affinity for HER2 than TAK-285, of which the interaction energy reached up to −65.8378 kcal/mol. Therefore, the preliminary analysis would serve as a modest spur to induce us to probe these novel 4-anilinoquinazoline compounds.

### Chemistry

(For experiment details please see Chemistry experimental section) Here we described our efforts to optimize the 6-salicyl-4-anilinoquinazoline series, mainly focusing on two aspects of modification: one is the replacement of substituents (include hydroxyl group) at the salicylic ring, and the other is the replacement of the -CH_2_-NH- linker with the carbonyl group between the quinazoline ring and the salicylic ring. A general synthetic approach was developed to prepare the 6- amino-4-anilinoquinazolines (compounds **7** and **8**) that can be easily generated and the corresponding process was displayed in Figure 4A [24]. Substitutions of 6-NH2 of the intermediates **7** and **8** by a series of salicylides with halogen groups led to 2-((4-(phenylamino)quinazolin-6-ylamino)methyl)phenol analogs **9–16** (Figure 4B). Furthermore, in order to explore how much the coplanar effect of the quinazoline core ring and salicylic ring contributed to compounds' activities, these corresponding salicylic amides **17–25** described also by Figure 4B were synthesized *via* a simple amidation pathway. Obviously seen that the flexible moiety (-CH_2_-NH-) was replaced by the amide structure (-CONH_2_-) in these compounds, and along with compounds **9–16**, all these compounds would be considered as probes used for the structure activity relationship (SAR) discussion.

Besides, we also noted the hydroxyl group of the salicylic ring and attempted to investigate what role it should play in the SAR study. We synthesized two new reference molecules **26** and **27**: one's hydroxyl group was replaced by an ethyoxyl, and the other possessed no hydroxyl group. As presented in Figure 4C, the salicylic acid dissolved in DMF, followed by reacting with C_2_H_5_I at 80°C, was converted to the intermediate 2-ethoxybenzoic acid and finally treated with thionyl chloride to yield compound **26**, and compound **27** from the starting material *para*-chlorobenzoic acid, was easily obtained by a simple amidation.

### EGFR/HER2 enzyme assay

**Figure 6:**
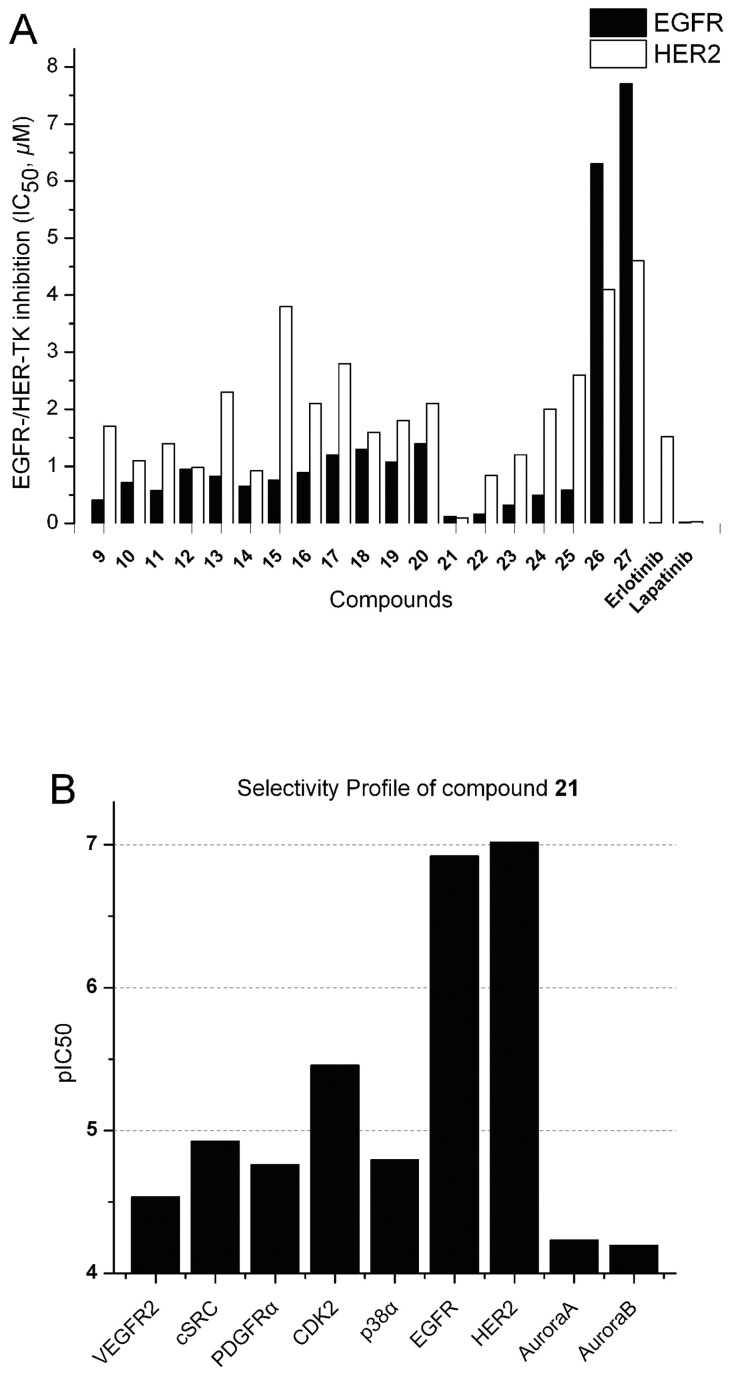
Results of kinase assays of compounds 9–27. (A) Inhibitory activity of compounds **9–27** against EGFR and HER2 protein kinases were represented as a bar graph. The black bar represented the IC_50_ values of all the compounds against EGFR tyrosine kinase *in vitro*, while the white one represented that of these compounds against HER2 tyrosine kinase. (B) Kinase selectivity profile of compound **21**. Potency is represented by pIC_50_.

The synthesized target compounds **9–27** were tested for their inhibitory activities toward the EGFR and HER2 tyrosine kinases [14] and their activity data were summarized in Table 1. As expected, most of all the synthesized compounds showed moderate to potent inhibitory activities for these two protein kinases. Of these 4-anilinoquinazoline compounds, compound **21** was screened and obtained as the most potent dual EGFR/HER2 inhibitors. In order to better displaying and comparing each other, these results were plotted as a bar graph presented in Figure 6A. It composed of two color bars in which the black bar represented EGFR inhibitory activities of all the compounds, while the white one represented HER2 inhibitory activities of all the compounds. Comparing the white one to the black one, the amplitude of activity variation depicted by the later of which IC_50_ values were in the range of 0.015−7 *μ*M, exhibited higher than that of the former of which the IC_50_ values were in the range of 0.03–4.6 *μ*M. Generally, compounds **9–20, 22–25** displayed less potency against HER2 inhibitory activities than EGFR inhibitory activities while the remaining three compounds **21**, **26**, **27** performed on the contrary. These results above demonstrated that 6-salicyl-4-anilinoquinazolines derived from compound **1** indeed preferred to bind to EGFR than HER2, with the exception of compound **21** or disruption of the salicyl ring (compounds **26** and **27**).

**Table 1:** Enzyme activities (IC_50_ a, *μ*M) of compounds 9–27 against human EGFR and HER2 kinases.

Compd.	EGFR	HER2
**9**	0.41±0.024	1.7±0.051
**10**	0.72±0.036	1.1±0.095
**11**	0.57±0.008	1.4±0.003
**12**	0.95±0.072	0.98±0.002
**13**	0.83±0.065	2.3±0.034
**14**	0.65±0.009	0.92±0.009
**15**	0.76±0.073	3.8±0.002
**16**	0.89±0.053	2.1±0.0087
**17**	1.2±0.071	2.8±0.0031
**18**	1.3±0.098	1.6±0.012
**19**	1.07±0.018	1.8±0.009
**20**	1.4±0.023	2.1±0.066
**21**	0.12±0.012	0.096±0.004
**22**	0.16±0.007	0.84±0.003
**23**	0.32±0.008	1.2±0.022
**24**	0.49±0.003	2±0.182
**25**	0.58±0.007	2.6±0.133
**26**	6.3±0.076	4.1±0.091
**27**	7.7±0.088	4.6±0.226
Erlotinib	0.015±0.003	1.52±0.002
Lapatinib	0.019±0.009	0.03±0.007

a Concentration to inhibit by 50% the phosphorylation of a polyglutamic acid/tyrosine random copolymer by EGFR enzyme (prepared from human A431 carcinoma cell vesicles by immunoaffinity chromatography) and HER2 enzyme (prepared from human MCF-7 breast cancer cell vesicles by immunoaffinity chromatography). Mean values in Micromolar, >1 determination.

Although the black bar could roughly correlate with the white one, there would be some attentions when comparing the variation tendency of EGFR to that of HER2. In part of compounds **9–16** with -CH_2_-NH- linker, the IC_50_ values of compounds against EGFR lowered than the level of 1 *μ*M while that against HER2 mostly exceeded to 1 *μ*M. Particularly, compound **14** that possessed 3,5-dichloro substituents performed better than the others, indicating that for inhibitory potency of EGFR and HER2 kinases, the chlorine atom as one substituent was likely to be superior to the bromine atom and disubstituted inhibitors were better than monosubstitutions. In addition, comparing these inhibitors **9–16** with -CH_2_-NH- linkers to those **17–25** containing -CO-NH- linkers, as was seen that that the flexible C-N bond was replaced by the rigid C = N bond can not radically influenced the corresponding activity, demonstrating that the coplanar effect between the quinazoline core and the salicylic ring was not important for the SAR of 6-salicyl −4-anilinoquinazolines.

Among compounds **17–27**, compound **21** showed the most potent inhibition toward EGFR and HER2, of which IC_50_ values can reach up to 0.12 and 0.096 *μ*M, respectively. Figure 6A showed that **21** could be considered as a turning point among these eleven analogs, before which compounds **17–20** with the Br atom on the aniline ring exhibited moderate activities against both of the two target proteins. Besides, it can be noted that the OH on the salicylic skeleton actually increased the inhibitory activity, comparing **17** to **27**. The deduction that the *pseudo* six-membered ring formed [25] through the intramolecular hydrogen bond between OH and O = C in the salicylic acid probably account for this issue above. The last one worth mentioning was that **22** with the methyl substituent is superior to **23** with the Cl atom and compounds with substitutions at the *meta* (**18–20, 25**) position showed less potent activities than those with substitutions at the *para* position (**17**, **21**, **23**, **24**). Thus, the primary factor for differences exhibited in the level of inhibitory activity of these compounds was determined by substituents on the salicylic ring and the trifluoromethyl of compound **21** has been identified as one of the most potent substituents on the salicylic ring.

### Antiproliferation assay

The target compounds were also evaluated in *in vitro* antiproliferation assays against three human cancer cells shown in Table 2: A549 (carcinomic human alveolar basal epithelial cell), MCF-7 (breast cancer, with Her2/neu protein overexpression) and A431 (overexpression of EGFR). As expected, due to different types of cancer cells correlated with EGFR/HER2 overexpression, the data revealed most compounds could perform better against MCF-7 cells and A431 cells than A549 cells. Among these compounds, compounds **7**, **9**, **12**, **13**, **19–24** could meanwhile inhibit MCF-7 and A431 at the level of IC_50_ values less than 1 *μ*M. In accord with the kinase inhibition, all the compounds, particularly from **17** to **25**, possessed the same trend with enzyme inhibitory activity. Strikingly, the most potent small molecule screened *via* these antiproliferation assays was still **21** (MCF-7 and A431, IC_50_  = 0.49 *µ*M and IC_50_  = 0.67 *µ*M, respectively). On the basis of the results above, in order to get the cellular activity profile of salicyl derivatives of 4-anilinoquinazoline against several known lapatinib-resistant cell lines, we select the most potent compound 21 and lapatinib as the positive control for futher biological evaluation as a potential dual EGFR/HER2 inhibitor. The screening results of **21** were obtained in Table 3 by means of three drug-resistant cancer cell lines that expressed or harbored genetic information with resistance to lapatinib. Comparing with the positive control, **21** showed a better profile of inhibiting the growth of these corresponding cancer cell lines. Particuarly it showed excellent growth inhibitory activity with IC_50_ of 0.77 µM in lapatinib-resistant H1781 cell line (IC_50_ (lapatinib)  = 2.1*µ*M). However, compound 21 as similar with lapatinib, exhibited weak inhibitory activity against H1975 cell line.

**Table 2:** *In vitro* antiproliferative activity (IC_50_, *μ*M) against three human tumor cell lines.

Compounds	Cellular IC_50_ (*µ*M)^a^		
	A549^b^	MCF-7^b^	A431^b^
**9**	2.37±0.089	0.77±0.008	0.98±0.003
**10**	1.93±0.071	0.84±0.004	1.03±0.027
**11**	2.13±0.128	0.90±0.018	0.87±0.009
**12**	1.82±0.062	1.07±0.016	1.14±0.036
**13**	2.17±0.049	0.92±0.012	1.26±0.051
**14**	1.59±0.038	0.80±0.005	0.92±0.062
**15**	2.12±0.022	0.81±0.033	0.96±0.005
**16**	1.65±0.094	0.99±0.006	1.32±0.126
**17**	1.59±0.035	1.21±0.029	1.46±0.027
**18**	0.80±0.004	1.23±0.035	1.62±0.008
**19**	1.77±0.056	1.12±0.087	1.28±0.013
**20**	1.80±0.167	1.31±0.029	1.39±0.023
**21**	1.64±0.027	0.49±0.018	0.67±0.002
**22**	1.39±0.054	0.61±0.005	0.74±0.006
**23**	1.36±0.016	0.55±0.007	0.84±0.009
**24**	1.30±0.012	0.47±0.004	0.63±0.004
**25**	1.69±0.078	0.74±0.013	0.88±0.011
**26**	1.58±0.093	0.82±0.082	0.76±0.002
**27**	1.78±0.034	1.29±0.076	1.48±0.034
**Erlotinib**	0.92±0.013	1.07±0.028	0.40±0.009
**Lapatinib**	1.12±0.022	0.21±0.003	0.33±0.005

a Antiproliferative activity was measured using the WST-1 assay. Values are the average of two independent experiments run in triplicate. Variation was generally 5%.

b Cancer cells kindly supplied by State Key Laboratory of Pharmaceutical. Biotechnology, Nanjing University; A549 (carcinomic human alveolar basal epithelial cell), MCF-7 (breast cancer, with Her2/neu protein overexpression) and A431 (overexpression of EGFR).

**Table 3:** *In Vitro* Cellular Activities of 21.

Tissue	Cell line^a^	Character^b^	IC_50_ (*µ*M)^c^	
			21	Lapatinib
Breast tigcancer	MDA- MB-453	HER-2++, PTEN deficiency	0.92±0.019	0.72±0.023
NSCLC^d^	H1781	HER-2 G776ins V_G/C	0.77±0.034	2.1±0.034
	H1975	EGFR L858R/ T790M	3.3±0.233	3.9±0.419

a Cancer cells were purchased from Nanjing KeyGen Biotech Co. Ltd, and subcultured by State Key Laboratory of Pharmaceutical Biotechnology, Nanjing University.

b The description was abstracted by the reference [27].

c Antiproliferative activity was measured using the WST-1 assay. Values are the average of two independent experiments run in triplicate. Variation was generally 5%.

d NSCLC: non-small-cell lung cancer.

### Kinase selectivity

Since 4-anilinoquinazoline analogs exhibited widely kinase inhibitions [26], the selectivity profile of compound **21** was assessed against several key kinases close to EGFR/HER2 (Figure 6B, for detailed data please see Table S1). The data confirmed the generally good selectivity profile of **21** but also identified some activity against CDK2, with an IC_50_ value of 3.5 *µ*M. In general, compound **21** bearing a triﬂuoromethyl group at the 4-position of the salicylic ring, which was proposed to fit the ATP binding pocket very well, showed more than 100-fold selectivity against major of other kinases screened.

### Molecular docking

Docking study was performed to fit compound **21** into the active center of the two protein-ligand complexes of the epidermal growth factor family (PDB code: 1M17 and 3RCD, respectively). The obtained results were presented in Figure 3C and 6F, which showed the optimal binding mode of compound **21** interacting with the 1M17 protein and the 3RCD protein, respectively. Compared with Figure 3A and 3B, Figure 3C displayed that although the binding mode of compound **21** to EGFR kinase protein seemed to differ from that of the control Erlotinib and compound **1**, the CDOCKER_INTERACTION_ENERGY of **21** was up to −49.3307 kcal/mol, lower than −47.3559 kcal/mol, that of compound **1**. When paying attention to Figure 3F, in addition to a similar manner to compound **1**, the binding mode of **21** to the HER2 protein was further optimized, in which multiple interactions that three key hydrogen bonds, one *Pi*-cation bond, and one *Pi*-Sigma bond could be provided clearly. Of three hydrogen bond interactions, two adjacent hydrogen bonds were formed between one fluorine atom of the trifluoromethyl moiety of **21** and two residues PHE731 and ALA730. The other hydrogen bond was formed between the guanidyl of LYS753 and the oxygen atom of the hydroxy group of **21**. One *Pi*-cation bond was located between the guanidyl of LYS753 and the aniline ring while the *Pi*-Sigma bond was formed by the quinazoline ring and VAL734. Insight into this picture, two amino acid residues SER783 and MET801 located in the hinge binding pocket seemed very important for the active conformation of compound **21**. Besides, the trifluoromethyl moiety, the hydroxy group, and the ketonic oxygen of **21** could contribute to stabilizing the binding complex. These results could provide a molecular level foundation for **21** as the most potent dual inhibitor.

## Conclusions

In summary, a series of 6-salicyl-4-anilinoquinazoline analogues **9–27** have been synthesized and showed modest to potent inhibitory potency against EGFR/HER2, with IC_50_ values ranging from 7.7 *µ*M to 0.096 *µ*M. Among these small molecules, compound **21** that possessed a *para* substituent of trifluoromethyl at the salicylic ring exhibited potent EGFR and HER2 kinase inhibitory activity with an IC_50_ of 0.12 *µ*M and 0.096 *µ*M, respectively, comparable to the control compounds Erlotinib and Lapatinib. The corresponding cellular activities of **21** against three lapatinib-resistant cancer cell lines were superior to the positive control lapatinib. Furthermore, molecular modeling confirmed this kind of novel binding modes of **21** to the EGFR/HER2 proteins, mainly due to the contribution of trifluoromethyl moiety. These wet results, along with molecular docking observations, could provide an important basis for further development of compound **21** as a potent tyrosine kinase inhibitor.

## Materials and Methods

All of the synthesized compounds were chemically characterized by thin layer chromatography (TLC), proton nuclear magnetic resonance (1H-NMR) and elemental microanalyses (CHN).^1^H NMR spectra were measured on a Bruker AV-300 or AV-500 spectrometer at 25°C and referenced to Me_4_Si. Chemical shifts are reported in ppm (*δ*) using the residual solvent line as internal standard. Splitting patterns are designed as *s*, singlet; *d*, doublet; *t*, triplet; *m*, multiplet. ESI-MS spectra were recorded on a Mariner System 5304 Mass spectrometer. Elemental analyses were performed on a CHN-O-Rapid instrument and were within ±0.4% of the theoretical values. Melting points were determined on a XT4 MP apparatus (Taike Corp., Beijing, China) and are as read. Analytical thin-layer chromatography (TLC) was performed on the glass-backed silica gel sheets (silica gel 60Å GF254). All compounds were detected using UV light (254 nm or 365 nm).

### General procedure for the preparation of compounds 7, 8

N4-(3-bromophenyl)quinazoline-4,6-diamine (**7**) or N4-(3-chlorophenyl)quinazoline-4,6-diamine (**8**) was prepared according to the literature procedure [2]. 5-Nitroanthranilonitrile **3** (4.89 g, 30 mmol, 1.0 equiv) was suspended in dimethylformamide dimethyl acetal (10 ml) and the mixture was refluxed for 2 h. The resulting mixture was cooled to room temperature for 2-3 h. The yellow precipitate that formed was filtered, washed with ethyl ether, and dried to give **4** (Yield: 80–90%). A mixture of **4** (2.18 g, 10 mmol, 1.0 equiv) and 3-chloroaniline (1.1 equiv) or 3-bromoaniline (1.1 equiv) were heated and stirred at reflux in acetic acid (20 ml) for 2 h. Aniline and acetic acid would be added into the reaction flask first and then slowly added **4** using the medicine spoon within one minute. This treatment is to prevent the formation of lumps between them. The yellow precipitate that formed was filtered hot, washed with hot acetic acid, diethyl ether, and dried to give the desired nitroquinazoline **5** or **6** (Yield: 80–90%).

6-Nitroquinazoline **6** (2.0 g, 6.65 mmol, 1.0 equiv), iron (2.5 g, 45 mmol, 6.7 equiv) and acetic acid (6 ml, 90 mmol, 13.5 equiv) were suspended in aqueous ethanol (180 ml, 77.8% v/v) and heated at reflux about 70–80°C for 5–6 h. Meantime this mixture would be stirred for one minute every half hour with a glass rod and the yellow solution would become reddish-brown slowly. The reaction mixture was cooled to room temperature and alkalinized by addition of concentrated ammonia (40 ml). Insoluble material was removed by filtration through Celite, and the filtrate was evaporated under reduced pressure. The resulting solid was extracted with ethyl acetate for column chromatography. Column chromatography was performed using silica gel (200–300 mesh) eluting with ethyl acetate and petroleum ether (3∶1, v/v) to give amine 6 (Yield: 50–60%). Mp 163–164°C;^ 1^H NMR (300 MHz, DMSO-*d*_6_, *δ* ppm): 4.08 (s, 2H, NH_2_), 6.93 (s, 1H), 7.13 (d, *J* = 7.86, 2H), 7.24 (dd, *J_1_*  = 11.13 Hz, *J_2_*  = 8.97 Hz, 1H), 7.33 (t, *J* = 8.06 Hz, 1H), 7.59 (d, *J* = 7.68 Hz, 1H), 7.78 (d, *J* = 8.97 Hz,1H), 7.94 (s,1H), 8.65 (s, 1H, NH). ESI-MS: 271.1 (C_14_H_11_ClN_4_, [M+H]^+^). Anal. Calcd for C_14_H_10_ClN_4_: C, 62.11%; H, 4.10%; N, 20.70%. Found: C, 61.93%; H, 4.36%; N, 21.07%. N4-(3-bromophenyl)quinazoline-4,6-diamine (**7**) was also prepared as described for **8**.

### General procedure for the synthesis of 9–16

Different substituent salicylal (100 mg) was added to a solution of the corresponding (**7** or **8**, 60 mg) in EtOH (20 ml). The mixture was stirred at 40–60°C for 4–6 h. In the next step, sodium borohydride (100 mg) was added into the liquid reactant, and the reaction mixture was stirred at room temperature for 1 d. The reaction mixture was evaporated under reduced pressure and the solid precipitated was dissolved with water (40 ml), extracted with ethyl acetate about three times. The extraction liquid was purified by a ﬂash chromatography with EtOAc/petroleum ether (2∶1, v/v) to give the target compounds as follows, which yields were between 60–80%.

### General procedure for the synthesis of 17–25

The starting materials (salicylic acids) for the synthesis of amides should be activated in the first procedure: acid (100 mg) and SOCl_2_ (6–10 ml) was mixed and stirred at reflux 80°C for 4 hours. The reaction mixture was cooled and evaporated to give reactive acyl chloride obtained as a kind of oil, which would be dissolved in ethyl acetate (5–6 ml) in the next step.

A solution of acyl chloride dissolved with ethyl acetate was added dropwise to compound **7** or **8** (0.5 mM) in ethyl acetate containing potassium carbonate (300 mg) at 0°C with constant stirring overnight. The reaction mixture was then poured in excess of diluted NaOH and extracted with EtOAc. The extraction liquid was purified by a ﬂash chromatography with EtOAc/petroleum ether (3∶1, v/v) to give these amides as follows: the yields were between 30–60%.

### General procedure for the synthesis of 26 and 27

Ethyl iodide (C_2_H_5_I, 2.4 ml) was added into the mixture containing salicylic acid (2 g) and potassium carbonate (4 g) in DMF (40–60 ml), and then the reaction mixture was stirred at 80°C for 6 h. The reaction mixture was poured in ice water and extracted with methylene dichloride. The solvent was removed *in vacuo* to obtain the corresponding salicylate as oil.

The total oil was added into the miscible liquid (NaOH, 4 g; EtOH, 30 ml; H_2_O, 50 ml) and was refluxed gently with stirring overnight. Adjusting pH value to 7 with hydrochloride, the 2-ethoxybenzoic acid was precipitated in the solution, filtered off to obtain a white solid (1.2 g). The next two steps of the preparation for **24** and **25** were the same as Figure 4B described above.

### 2-((4-(3-bromophenylamino)quinazolin-6-ylamino)methyl)-4-chlorophenol (*9*)

Mp: 230–232°C; ^1^H NMR (500 MHz, DMSO-d6, *δ* ppm): 4.45 (s, 2H, CH_2_), 6.48 (s, 1H), 6.87 (d, *J* = 8.5 Hz, 1H), 7.13 (d, *J* = 8.5 Hz, 1H), 7.26 (d, *J* = 8.0 Hz, 1H), 7.31 (s, 2H), 7.33–7.41 (m, 2H), 7.57 (d, *J* = 8.5 Hz, 1H), 7.89 (d, *J* = 8.0 Hz, 1H), 8.16 (s, 1H, NH), 8.40(s, 1H), 9.40 (s, 1H, NHCH_2_), 9.92 (s, 1H, OH).^ 13^C NMR (100 MHz, DMSO-d6, *δ* ppm): 158.37, 155.34, 151.93, 148.87, 144.59, 141.36, 131.21, 130.15, 129.74, 128.96, 127.13, 126.04, 125.17, 124.33, 122.63, 121.75, 117.66, 116.22, 114.87, 109.39, 44.57. ESI-MS: 456.7 (C_21_H_17_BrClN_4_O, [M+H]^+^). Anal. Calcd for C_21_H_16_BrClN_4_O: C, 55.34%; H, 3.54%; N, 12.29%. Found: C, 55.67%; H, 3.81%; N, 11.99%.

### 2-((4-(3-bromophenylamino)quinazolin-6-ylamino)methyl)-4,6-dichlorophenol (*10*)

Mp: 214–216°C; ^1^H NMR (500 MHz, DMSO-d6, *δ* ppm): 4.45 (d, *J* = 5.5 Hz, 2H, CH_2_), 6.58 (s, 1H), 7.26 (d, *J* = 7.5 Hz, 1H), 7.30 (s, 2H), 7.32–7.37 (m, 2H), 7.42 (s, 1H), 7.58 (d, *J* = 9.0 Hz, 1H), 7.88 (d, *J* = 8.0 Hz, 1H), 8.15 (s, 1H, NH), 8.39 (s, 1H), 9.39 (s, 1H, NHCH_2_), 9.78 (s, 1H, OH).^ 13^C NMR (100 MHz, DMSO-d6, *δ* ppm): 158.37, 154.28, 151.93, 148.87, 144.59, 141.36, 131.21, 130.15, 128.99, 128.87, 128.61, 126.04, 125.17, 123.96, 122.63, 121.75, 119.81, 117.66, 114.87, 109.39, 46.78. ESI-MS: 491.2 (C_21_H_16_BrCl_2_N4O, [M+H]^+^). Anal. Calcd for C_21_H_15_BrCl_2_N4O: C, 51.46%; H, 3.08%; N, 11.43%. Found: C, 51.74%; H, 3.12%; N, 11.62%.

### 4-bromo-2-((4-(3-bromophenylamino)quinazolin-6-ylamino)methyl)phenol (*11*)

Mp: 189–191°C; ^1^H NMR (500 MHz, DMSO-d6, *δ* ppm): 4.39 (s, 2H, CH_2_), 6.49 (s, 1H), 6.83 (d, *J* = 8.5Hz, 1H), 7.24–7.28 (m, 2H), 7.31–7.35 (m, 2H), 7.38 (d, *J* = 9.0Hz, 1H), 7.44 (s, 1H), 7.57 (d, *J* = 9.0Hz, 1H), 7.89 (d, *J* = 8.0Hz, 1H), 8.16 (s, 1H, NH), 8.39 (s, 1H), 9.41 (s, 1H, NHCH_2_), 9.95 (s, 1H, OH).^ 13^C NMR (100 MHz, DMSO-d6, *δ* ppm): 158.37, 155.06, 151.93, 148.87, 144.59, 141.36, 132.28, 131.21, 130.83, 130.15, 128.47, 126.04, 125.17, 122.63, 121.75, 118.62, 117.66, 114.87, 110.66, 109.39, 44.57. ESI-MS: 501.2 (C_21_H_17_Br_2_N_4_O, [M+H]^+^). Anal. Calcd for C_21_H_16_Br_2_N_4_O: C, 50.43%; H, 3.22%; N, 11.20%. Found: C, 50.51%; H, 3.29%; N, 11.12%.

### 2,4-dibromo-6-((4-(3-bromophenylamino)quinazolin-6-ylamino)methyl)phenol (*12*)

Mp: 169–171°C; ^1^H NMR (300 MHz, DMSO-d6, *δ* ppm): 4.46 (d, *J* = 5.7Hz, 2H, CH_2_), 6.62 (s, 1H), 7.26–7.37 (m, 4H), 7.46 (s, 1H), 7.59 (d, *J* = 9.0 Hz, 1H), 7.64 (s, 1H), 7.86 (d, *J* = 8.1 Hz, 1H), 8.14 (s, 1H, NH), 8.40 (s, 1H), 9.47 (s, 1H, NHCH_2_), 9.68 (s, 1H, OH).^ 13^C NMR (100 MHz, DMSO-d6, *δ* ppm): 158.37, 155.34, 151.93, 148.87, 144.59, 141.36, 133.29, 131.57, 131.21, 130.15, 129.54, 126.04, 125.17, 122.63, 121.75, 117.66, 114.87, 112.33, 109.88, 109.39, 46.78. ESI-MS: 580.1 (C_21_H_16_Br_3_N_4_O, [M+H]^+^). Anal. Calcd for C_21_H_15_Br_3_N_4_O: C, 43.56%; H, 2.61%; N, 9.68%. Found: C, 43.85%; H, 2.83%; N, 9.47%.

### 4-chloro-2-((4-(3-chlorophenylamino)quinazolin-6-ylamino)methyl)phenol (*13*)

Mp: 238–240°C; ^1^H NMR (300 MHz, DMSO-d6, *δ* ppm): 4.38 (d, *J* = 5.7 Hz, 2H, CH_2_), 6.51 (s, 1H), 6.87 (d, *J* = 9.0 Hz, 1H), 7.13 (dd, *J_1_*  = 8.4 Hz, *J_2_*  = 8.7 Hz, 2H), 7.31 (s, 2H), 7.36–7.42 (m, 2H), 7.57 (d, *J* = 8.7 Hz,1H), 7.82 (d, *J* = 8.4 Hz, 1H), 8.05 (s, 1H, NH), 8.39 (s, 1H), 9.43 (s, 1H, NHCH_2_), 9.94 (s, 1H, OH).^ 13^C NMR (100 MHz, DMSO-d6, *δ* ppm): 158.37, 155.34, 151.93, 148.87, 144.61, 141.36, 134.52, 131.21, 130.67, 129.81, 128.62, 127.92, 124.13, 122.18, 120.76, 120.21, 117.66, 116.22, 114.87, 109.39, 44.57. ESI-MS: 412.3 (C_21_H_17_Cl_2_N_4_O, [M+H]^+^). Anal. Calcd for C_21_H_16_Cl_2_N_4_O: C, 61.33%; H, 3.92%; N, 13.62%. Found: C, 61.14%; H, 3.82%; N, 13.56%.

### 2,4-dichloro-6-((4-(3-chlorophenylamino)quinazolin-6-ylamino)methyl)phenol (*14*)

Mp: 211–213°C; ^1^H NMR (300 MHz, DMSO-d6, *δ* ppm): 4.45 (d, *J* = 5.7 Hz, 2H, CH_2_), 6.6 (s, 1H), 7.13 (d, *J* = 7.5 Hz, 1H), 7.30 (s, 2H), 7.34–7.42 (m, 3H), 7.59 (d, *J* = 9.0 Hz, 1H), 7.81 (d, *J* = 8.1 Hz, 1H), 8.03 (s, 1H, NH), 8.40 (s, 1H), 9.44 (s, 1H, NHCH_2_), 9.80 (s, 1H, OH). ^13^C NMR (100 MHz, DMSO-d6, *δ* ppm): 158.37, 154.37, 151.93, 148.87, 144.61, 141.36, 134.52, 131.21, 130.67, 128.99, 128.79, 128.61, 123.99, 122.18, 120.76, 120.21, 119.61, 117.66, 114.87, 109.39, 46.78. ESI-MS: 446.7 (C_21_H_16_Cl_3_N_4_O, [M+H]^+^). Anal. Calcd for C_21_H_15_Cl_3_N_4_O: C, 56.59%; H, 3.39%; N, 12.57%. Found: C, 56.41%; H, 3.78%; N, 12.64%.

### 4-bromo-2-((4-(3-chlorophenylamino)quinazolin-6-ylamino)methyl)phenol (*15*)

Mp: 227–229°C; ^1^H NMR (300 MHz, DMSO-d6, *δ* ppm): 4.39 (d, *J* = 5.4 Hz, 2H, CH_2_), 6.52 (s, 1H), 6.83 (d, *J* = 8.7 Hz, 1H), 7.13 (dd, *J_1_*  = 7.8 Hz, *J_2_*  = 8.1 Hz, 1H), 7.25 (dd, *J_1_*  = 9.0 Hz, *J_2_*  = 9.0 Hz, 1H), 7.32 (s, 1H), 7.37–7.45 (m, 3H), 7.58 (d, *J* = 9.0 Hz, 1H), 7.84 (d, *J* = 9.0 Hz, 1H), 8.06 (s, 1H, NH), 8.40 (s, 1H), 9.44 (s, 1H, NHCH_2_), 9.99 (s, 1H, OH).^ 13^C NMR (100 MHz, DMSO-d6, *δ* ppm): 158.37, 154.86, 151.93, 148.87, 144.53, 141.36, 134.52, 132.08, 131.21, 130.73, 130.67, 128.47, 122.18, 120.76, 120.21, 118.62, 117.66, 114.87, 110.66, 109.39, 44.57. ESI-MS: 455.0 (C_21_H_17_BrClN_4_O, [M+H]^+^). Anal. Calcd for C_21_H_16_BrClN_4_O: C, 55.34%; H, 3.54%; N, 12.29%. Found: C, 55.51%; H, 3.65%; N, 12.38%.

### 2,4-dibromo-6-((4-(3-chlorophenylamino)quinazolin-6-ylamino)methyl)phenol (*16*)

Mp: 207–209°C; ^1^H NMR (300 MHz, DMSO-d6, *δ* ppm): 4.46 (d, *J* = 5.7 Hz, 2H, CH_2_), 6.65 (s, 1H), 7.16 (d, *J* = 6.6 Hz, 1H), 7.32 (s, 1H), 7.37 (dd, *J*_1_  = 9.0 Hz, *J_2_*  = 6.0 Hz, 1H), 7.42 (d, *J = *8.1 Hz, 1H), 7.46 (s, 1H), 7.59 (d, *J* = 9 Hz, 1H), 7.65 (d, *J* = 3 Hz, 1H), 7.8 (d, *J* = 9.0 Hz, 1H), 8.02 (s, 1H, NH), 8.43 (s, 1H), 9.57 (s, 1H, NHCH_2_), 9.67 (s, 1H, OH).^ 13^C NMR (100 MHz, DMSO-d6, *δ* ppm): 158.37, 154.42, 151.93, 148.87, 144.61, 141.36, 134.52, 133.29, 131.57, 131.21, 130.67, 129.54, 122.18, 120.76, 120.21, 117.66, 114.87, 112.33, 109.88, 109.39, 46.78. ESI-MS: 535.6 (C_21_H_16_Br_2_ClN_4_O, [M+H]^+^). Anal. Calcd for C_21_H_15_Br_2_ClN_4_O: C, 47.18%; H, 2.83%; N, 10.48%. Found: C, 47.32%; H, 2.96%; N, 10.65%.

### N-(4-(3-bromophenylamino)quinazolin-6-yl)-4-chloro-2-hydroxybenzamide (*17*)

Mp: 274–277°C; ^1^H NMR (300 MHz, DMSO-d6, *δ* ppm): 7.09 (d, *J* = 7.8 Hz, 2H), 7.29–7.39 (m, 2H), 7.83–7.90 (m, 2H), 8.03 (d, *J* = 9.0 Hz, 1H), 8.10 (d, *J* = 9.0 Hz, 1H), 8.20 (s, 1H, NH), 8.63 (s, 1H), 8.78 (s, 1H), 9.94 (s, 1H, OH), 10.71 (s, 1H), 12.14 (s, 1H, NHCO). ^13^C NMR (100 MHz, DMSO-d6, *δ* ppm): 169.13, 158.94, 158.37, 151.93, 148.87, 144.59, 137.41, 134.72, 130.97, 130.15, 129.85, 128.09, 126.04, 125.17, 122.63, 121.75, 119.61, 117.31, 116.48, 114.87, 113.62. ESI-MS: 470.7 (C_21_H_15_BrClN_4_O_2_, [[M+H]^+^). Anal. Calcd for C_21_H_14_BrClN_4_O_2_: C, 53.70%; H, 3.00%; N, 11.93%. Found: C, 53.83%; H, 3.07%; N, 12.01%.

### 5-bromo-N-(4-(3-bromophenylamino)quinazolin-6-yl)-2-hydroxybenzamide (*18*)

Mp: 297–299°C; ^1^H NMR (300 MHz, DMSO-d6, *δ* ppm): 7.02 (d, *J* = 8.7 Hz, 1H), 7.29–7.39 (m, 2H), 7.63 (d, *J* = 6.9 Hz, 1H), 7.87 (t, *J* = 6.0 Hz, 2H), 8.10–8.14 (m, 2H), 8.20 (s, 1H, NH), 8.63 (s, 1H), 8.77 (s, 1H), 9.94 (s, 1H, OH), 10.76 (s, 1H), 11.95 (s, 1H, NHCO). ^13^C NMR (100 MHz, DMSO-d6, *δ* ppm): 167.87, 160.28, 158.37, 151.93, 148.87, 145.53, 136.18, 134.72, 132.58, 130.15, 129.85, 128.09, 126.04, 125.17, 122.63, 121.75, 118.93, 117.34, 114.87, 113.62, 111.73. ESI-MS: 515.2 (C_21_H_15_Br_2_N4O_2_, [M+H]^+^). Anal. Calcd for C_21_H_14_Br_2_N4O_2_: C, 49.05%; H, 2.74%; N, 10.90%. Found: C, 49.17%; H, 2.94%; N, 10.66%.

### N-(4-(3-bromophenylamino)quinazolin-6-yl)-5-chloro-2-hydroxybenzamide (*19*)

Mp: 304–306°C; ^1^H NMR (300 MHz, DMSO-d6, *δ* ppm): 7.08 (d, *J* = 8.7 Hz, 1H), 7.32–7.39 (m, 2H), 7.52 (d, *J* = 7.5 Hz, 1H), 7.87 (t, *J* = 6.0 Hz, 2H), 8.03 (s, 1H), 8.11 (d, *J* = 8.1 Hz, 1H), 8.20 (s, 1H, NH), 8.62 (s, 1H), 8.78 (s, 1H), 9.94 (s, 1H, OH), 10.75 (s, 1H), 11.95 (s, 1H, NHCO).^ 13^C NMR (100 MHz, DMSO-d6, *δ* ppm): 167.87, 158.93, 158.37, 151.93, 148.87, 144.59, 135.14, 134.72, 130.15, 129.85, 128.83, 128.09, 126.57, 126.04, 125.17, 122.63, 121.75, 118.16, 117.75, 114.87, 113.62. ESI-MS: 470.7 (C_21_H_15_BrClN_4_O_2_, [M+H]^+^). Anal. Calcd for C_21_H_14_BrClN_4_O_2_: C, 53.70%; H, 3.00%; N, 11.93%. Found: C, 53.74%; H, 3.09%; N, 11.63%.

### 3,5-dibromo-N-(4-(3-bromophenylamino)quinazolin-6-yl)-2-hydroxybenzamide (*20*)

Mp: 274–276°C; ^1^H NMR (300 MHz, DMSO-d6, *δ* ppm): 7.04 (s, 1H), 7.29–7.42 (m, 2H), 7.91–7.83 (m, 2H), 8.08–8.13 (m, 2H), 8.21 (s, 1H, NH), 8.63 (s, 1H), 8.76 (s, 1H), 9.92 (s, 1H, OH), 10.70 (s, 1H), 12.00 (s, 1H, NHCO).^ 13^C NMR (100 MHz, DMSO-d6, *δ* ppm): 167.34, 158.37, 158.26, 151.93, 148.87, 144.59, 140.05, 134.72, 131.07, 130.15, 129.85, 128.09, 126.04, 125.17, 122.63, 121.75, 118.63, 114.87, 113.62, 111.36, 110.33. ESI-MS: 594.1 (C_21_H_14_Br_3_N_4_O_2_, [M+H]^+^). Anal. Calcd for C_21_H_13_Br_3_N_4_O_2_: C, 42.53%; H, 2.21%; N, 9.45%. Found: C, 42.73%; H, 2.27%; N, 9.64%.

### N-(4-(3-bromophenylamino)quinazolin-6-yl)-2-hydroxy-4-(trifluoromethyl)benzamide (*21*)

Mp: 289–291°C; ^1^H NMR (300 MHz, DMSO-d6, *δ* ppm): 7.26–7.39 (m, 5H), 7.80–7.91 (m, 2H), 8.10 (d, *J* = 9.0 Hz, 2H), 8.19 (s, 1H, NH), 8.63 (s, 1H), 8.82 (s, 1H), 9.95 (s, 1H, OH), 10.86 (s, 1H, NHCO). ^13^C NMR (100 MHz, DMSO-d6, *δ* ppm): 169.13, 161.87, 158.37, 151.93, 148.87, 144.59, 134.72, 133.17, 130.15, 129.85, 129.54, 128.09, 126.04, 125.17, 124.69, 124.14, 122.63, 121.66, 121.75, 117.46, 114.87, 113.62. ESI-MS: 504.3 (C_22_H_14_BrF_3_N_4_O_2_, [M+H]^+^). Anal. Calcd for C_22_H_14_BrF_3_N_4_O_2_: C, 52.50%; H, 2.80%; N, 11.13%. Found: C, 52.74%; H, 2.89%; N, 11.32%.

### 4.5.14. N-(4-(3-chlorophenylamino)quinazolin-6-yl)-2-hydroxybenzamide (*22*)

Mp: 117–120°C; ^1^H NMR (300 MHz, DMSO-d6, *δ* ppm): 7.23–7.39 (m, 3H), 7.46–7.50 (m, 2H), 7.84–7.95 (m, 3H), 8.09 (s, 1H, NH), 8.12 (d, *J* = 8.94 Hz, 1H), 8.63 (s, 1H), 8.78 (s, 1H), 9.93 (s, 1H, OH), 10.73 (s, 1H), 11.84 (s, 1H, NHCO).^ 13^C NMR (100 MHz, DMSO-d6, *δ* ppm): 169.13, 160.98, 158.37, 151.93, 148.87, 144.61, 134.52, 135.77, 134.72, 130.07, 129.85, 129.14, 128.09, 122.18, 120.76, 120.21, 119.88, 117.13, 115.95, 114.87, 113.62. ESI-MS: 391.8 (C_21_H_16_ClN_4_O_2_, [M+H]^+^). Anal. Calcd for C_21_H_15_ClN_4_O_2_: C, 64.54%; H, 3.87%; N, 14.34%. Found: C, 64.73%; H, 4.12%; N, 14.51%.

### 4-chloro-N-(4-(3-chlorophenylamino)quinazolin-6-yl)-2-hydroxybenzamide (*23*)

Mp: 211–213°C; ^1^H NMR (300 MHz, DMSO-d6, *δ* ppm): 7.07 (d, *J* = 2.01 Hz, 1H), 7.40 (t, *J* = 8.04 Hz, 1H), 7.71–7.88 (m, 4H), 7.95–8.01 (m, 2H), 8.59 (s, 1H), 8.78 (s, 1H), 9.95 (s, 1H, OH), 10.55 (s, 1H), 10.81 (s, 1H, NHCO).^ 13^C NMR (100 MHz, DMSO-d6, *δ* ppm): 169.13, 158.94, 158.37, 151.93, 148.87, 144.61, 137.41, 134.72, 134.52, 130.67, 130.07, 129.85, 128.09, 122.18, 120.76, 120.21, 119.61, 117.31, 116.48, 114.87, 113.62. ESI-MS: 426.3 (C_21_H_15_Cl_2_N_4_O_2_, [M+H]^+^). Anal. Calcd for C_21_H_14_Cl_2_N_4_O_2_: C, 59.31%; H, 3.32%; N, 13.17%. Found: C, 59.27%; H, 3.41%; N, 13.19%.

### N-(4-(3-chlorophenylamino)quinazolin-6-yl)-2-hydroxy-4-methylbenzamide (*24*)

Mp: 257–258°C; ^1^H NMR (500 MHz, DMSO-d6, *δ* ppm): 2.35 (s, 3H, CH_3_), 6.84 (d, *J* = 9.5 Hz, 1H), 7.19 (d, *J* = 6.5 Hz, 1H), 7.43 (t, *J* = 4.25 Hz, 1H), 7.84 (t, *J* = 4.75 Hz, 2H), 7.97 (d, *J* = 8.0 Hz, 1H*),*8.08 (s, 1H, NH), 8.13 (d, *J* = 9.0 Hz, 1H), 8.65 (s, 1H), 8.78 (s, 1H), 10.02 (s, 1H, OH), 10.69 (s, 1H), 11.90 (s, 1H, NHCO). ^13^C NMR (100 MHz, DMSO-d6, *δ* ppm): 169.13, 160.69, 158.37, 151.93, 148.87, 144.61, 142.57, 134.72, 134.52, 130.67, 129.85, 128.56, 128.09, 122.18, 120.92, 120.76, 120.21, 119.74, 115.96, 114.87, 113.62, 24.71. ESI-MS: 405.9 (C_22_H_18_ClN_4_O_2_, [M+H]^+^). Anal. Calcd for C_22_H_17_ClN_4_O_2_: C, 65.27%; H, 4.23%; N, 13.84%. Found: C, 65.53%; H, 4.21%; N, 13.53%.

### 5-bromo-N-(4-(3-chlorophenylamino)quinazolin-6-yl)-2-hydroxybenzamide (*25*)

Mp: 299–301°C; ^1^H NMR (300 MHz, DMSO-d6, *δ* ppm): 7.01 (d, *J* = 9.0 Hz, 1H), 7.16 (d, *J* = 7.8 Hz, 1H), 7.41 (t, *J* = 4.1 Hz, 1H), 7.59–7.63 (m, 1H), 7.82 (t, *J* = 4.5 Hz, 2H), 8.07 (s, 1H, NH), 8.11–8.14 (m, 2H), 8.61 (s, 1H), 8.76 (s, 1H), 9.34 (s, 1H, OH), 10.73 (s, 1H), 11.94 (s, 1H, NHCO).^ 13^C NMR (100 MHz, DMSO-d6, *δ* ppm): 167.87, 160.28, 158.37, 151.93, 148.87, 144.61, 136.18, 134.72, 134.52, 132.58, 130.67, 129.85, 128.09, 122.18, 120.76, 120.21, 118.93, 117.34, 114.87, 113.62, 111.73. ESI-MS: 470.7 (C_21_H_15_BrClN_4_O_2_, [M+H]^+^). Anal. Calcd for C_21_H_14_BrClN_4_O_2_: C, 53.70%; H, 3.00%; N, 11.93%. Found: C, 53.79%; H, 3.12%; N, 11.74%.

### N-(4-(3-chlorophenylamino)quinazolin-6-yl)-2-ethoxybenzamide (*26*)

Mp: 111–113°C; ^1^H NMR (300 MHz, DMSO-d6, *δ* ppm): 4.01–4.07 (m, 5H, OC_2_H_5_), 7.10–7.14 (m, 3H), 7.46–7.54 (m, 2H), 7.65–7.86 (m, 5H), 8.07 (s, 1H, NH), 8.56 (s, 1H), 8.81 (s, 1H), 10.71 (s, 1H, NHCO).^ 13^C NMR (100 MHz, DMSO-d6, *δ* ppm): 166.47, 158.37, 157.36, 151.93, 148.87, 144.61, 134.72, 134.52, 133.91, 131.99, 130.67, 129.85, 128.09, 124.94, 122.18, 122.03, 120.76, 120.21, 115.82, 114.87, 113.62, 66.54, 18.93. ESI-MS: 419.9 (C_23_H_20_ClN_4_O, [M+H]^+^). Anal. Calcd for C_23_H_19_ClN_4_O: C, 65.95%; H, 4.57%; N, 13.38%. Found: C, 65.81%; H, 4.76%; N, 13.21%.

### N-(4-(3-bromophenylamino)quinazolin-6-yl)-4-chlorobenzamide (*27*)

Mp: 230–232°C; ^1^H NMR (300 MHz, DMSO-d6, *δ* ppm): 7.07 (d, *J* = 8.4 Hz, 2H), 7.30–7.43 (m, 3H), 7.72–7.86 (m, 2H), 8.04 (d, *J* = 8.4 Hz, 1H), 8.11 (d, *J* = 8.4 Hz, 1H), 8.18 (s, 1H, NH), 8.63 (s, 1H), 8.78 (s, 1H), 10.82 (s, 1H), 11.54 (s, 1H, NHCO).^ 13^C NMR (100 MHz, DMSO-d6, *δ* ppm): 167.56, 158.37, 151.93, 148.87, 144.59, 137.43, 134.52, 130.15, 129.85, 128.82, 128.22, 128.09, 126.04, 125.17, 122.63, 121.75, 114.87, 113.62. ESI-MS: 454.7 (C_21_H_15_BrClN_4_O, [M+H]^+^). Anal. Calcd for C_21_H_14_BrClN_4_O: C, 55.59%; H, 3.11%; N, 12.35%. Found: C, 55.72%; H, 3.18%; N, 12.14%.

## Supporting Information

Text S1 **Biological experimental data of the synthesized compounds and molecular docking study.**
(DOC)
