# Supplementary material for: Optimization of Substituted 6-Salicyl-4-Anilinoquinazoline Derivatives as Dual EGFR/HER2 Tyrosine Kinase Inhibitors
Source: PLoS One. 2013 Aug 1;8(8):e69427. doi: 10.1371/journal.pone.0069427 (PMC3731256; doi:10.1371/journal.pone.0069427)
Supplement: Text S1 — Biological experimental data of the synthesized compounds and molecular docking study. (DOC) [file pone.0069427.s001.doc]

***Electronic supplementary information (ESI)***

**Optimization of Substituted** **6-****Salicyl-****4-Anilinoquinazoline Derivatives as Dual EGFR/HER2 Tyrosine Kinase Inhibitors**

Dong-Dong Li ^1+^, Ya-Juan Qin ^1+^, Jian Sun ^1^, Jing-Ran Li ^1^, Fei Fang ^1^, Qian-Ru Du^1^, Yong Qian ^1^, Hai-Bin Gong ^2*^, Hai-Liang Zhu ^1*^

*^1^ State Key Laboratory of Pharmaceutical Biotechnology, Nanjing University, Nanjing, P. R. China.*

*^2^ Xuzhou Central Hospital, Xuzhou, P. R. China.*

*Tel.: +86-25-8359 2572; Fax: 0086+25+8359 2672; E-mail address: zhuhl@nju.edu.cn.*

**Contents**

Table S1**…………………………………………………………………………...**S2

Biological assay protocols**………………………………………………………...**S2

Molecular docking study**…………………………………………………….........**S4

References**………………………………………………………………………...**S5

**Table S1.** The selectivity proﬁle of compound **21**

| Kinase | IC_50_ ^a^ | pIC_50_ ^b^ |
| --- | --- | --- |
| VEGFR2 | 29.2±0.98 | 4.53 |
| cSRC | 11.9±0.78 | 4.92 |
| PDGFRα | 17.3±0.06 | 4.76 |
| CDK2 | 3.5±0.05 | 5.46 |
| p38α | 16.1±0.37 | 4.79 |
| EGFR | 0.12±0.012 | 6.92 |
| HER2 | 0.096±0.004 | 7.02 |
| Aurora A | 58.4±0.78 | 4.23 |
| Aurora B | 63.7±1.02 | 4.20 |

^a^ µM, ATP concentration = K_m_, _ATP_ of the tested kinase.

^b^ The IC_50_ values of the compounds against kinases were converted into pIC_50_ values by using the online calculator.(http://www.sanjeevslab.org/tools-IC50.html).

**Biological assay protocols**

**HER2 and EGFR kinase assay**

The cytoplasmic domain (amino acids 676-1255) of human HER2 and the cytoplasmic domain (amino acids 669-1210) of human EGFR were expressed as N-terminal peptide (DYKDDDD)-tagged protein using a baculovirus expression system. The expressed HER2 kinase and EGFR kinase were purified by anti-FLAG M2 affinity gel (Sigma-Aldrich, USA). The HER2 and EGFR kinase assays were performed using radiolabeled [γ-^32^P] ATP (GEHealthcare, USA) in 96-well plates. The kinase reactions were performed in 50 mmol/L Tris-HCl, pH7.5, 5 mmol/L MnCl_2_, 0.01% Tween-20 and 2 mmol/L DTT containing 0.9 *μ*Ci of [γ-^32^P] ATP per reaction, 50 *μ*mol/L ATP, 5 *μ*g/mL poly-Glu-Tyr (4:1) and 0.25 *μ*g/mL of purified Her2 or EGFR cytoplasmic domain in a total volume of 50 *μ*L. To measure the IC_50_ value for enzyme inhibition, compounds were incubated with the enzyme for 5 minutes prior to the reaction at room temperature. Kinase reactions were initiated by adding ATP. After the kinase reaction incubated for 10 minutes at room temperature, reactions were terminated by the addition of 10% (final concentration) trichloroacetic acid. The [γ-^32^P]-phosphorylated proteins were filtered in a harvest plate (Millipore, USA) with a cell harvester (PerkinElmer, USA) and washed free of [γ-^32^P] ATP with 3% phosphoric acid. The plates were dried, followed by the addition of 25 *μ*L of MicroScintO (PerkinElmer, USA). Radioactivity was counted by a Topcount scintillation counter (PerkinElmer, USA). IC_50_ values and 95% confidence intervals were calculated by nonlinear regression analysis.

**Cell proliferation assay**

WST-1 is better than MTT for analyzing cell proliferation, because it can be reduced to soluble formazan by dehydrogenase in mitochondria and has little toxicity to cells. Cell proliferation was determined using WST-1 dye (Beyotime Inst Biotech, China) according to manufacture’s instructions. Brieﬂy, 5 × 10^3^ cells per well were seeded in a 96-well plate, grown at 37 ℃ for 24 h, and then placed in serum-starved conditions for a further 6 h. Subsequently, cells were treated with compounds (Table 1) at increasing concentrations in the presence of 10% FBS for 24 or 48h. After 10 µL WST-1 dye was added to each well, cells were incubated at 37 ℃ for 2 h and the absorbance was ﬁnally determined at 450 nm using a microplate reader.

**Kinase selectivity profiling** [1]

**Serine/threonine kinase proﬁling by IC_50_ measurement**

Assays for 4 serine/threonine kinases using radio labeled [γ-^32^P] ATP (GE Healthcare, Piscataway, NJ) were performed in 96 well plates. Mitogen-activated protein kinase p38α (p38α), was expressed as N-terminal FLAG-tagged protein using a baculovirus expression system. Aurora-A and -B were expressed as N-terminal 6xHis tagged protein using a baculovirus expression system. CDK2/CycA was expressed as C-terminal 6His-tagged CDK2, and N-terminal GST-tagged Cyclin A protein using a baculovirus expression system.

The reaction conditions were optimized for each kinase: p38α (100 ng/well of enzyme, 1 *μ*g/well of MBP (Wako Pure Chemical Ind., Osaka, Japan), 0.1 *μ*Ci/well of [γ-^32^P] ATP, 60 min reaction at 30 ℃); Aurora-A and -B (50 ng/well of enzyme, 30 *μ*M of Aurora substrate peptide, 0.2 *μ*Ci/well of [γ-^32^P] ATP, 60 min reaction at room temperature); CDK2/CycA (1.8 mUnits/well of enzyme, 1 *μ*g/well of Histone H1 (Calbiochem), 0.2 *μ*Ci/well of [γ-^32^P] ATP, 20 min reaction at room temperature).

All the four enzyme reactions were performed in 25 mM HEPES, pH 7.5, 10 mM magnesium acetate, 1 mM dithiothreitol and 0.5 *μ*M ATP containing optimized concentration of enzyme, substrate and radiolabeled ATP as described above in a total volume of 50 *μ*l. Prior to the kinase reaction, compound and enzyme were incubated for 5 min at reaction temperature as described above. The kinase reactions were initiated by adding ATP. After the reaction period as described above, the reactions were terminated by the addition of 10% (ﬁnal concentration) trichloroacetic acid. The [γ-^32^P]-phosphorylated proteins were ﬁltrated in Harvest Plate (Millipore Corp.) with a Cell Harvester (PerkinElmer) and then free of [γ-^32^P] ATP was washed out with 3% phosphoric acid. The plates were dried, followed by the addition of 40 *μ*l of MicroScint0 (PerkinElmer). The radioactivity was counted by a TopCount scintillation counter (PerkinElmer).

**Tyrosine kinase proﬁling by IC_50_ measurement**

The cytoplasmic domain of vascular endothelial growth factor receptor 2 (VEGFR2), platelet-derived growth factor receptor α (PDGFRα), cSRC were purchased from Millipore Corp. Assays for 3 tyrosine kinases were performed in 384 well plates using the Alphascreen® system (PerkinElmer) at room temperature. Enzyme reactions were performed in 50 mM Tris–HCl, pH7.5, 5 mM MnCl_2_, 5 mM MgCl_2_, 0.01% Tween-20, 2 mM dithiothreitol and 0.1 *μ*g/ml biotinylated poly-GluTyr (4:1) containing optimized concentration of enzyme and ATP as described below.

Prior to the kinase reaction, compound and enzyme were incubated for 5 min at room temperature. The reactions were initiated by adding ATP. After the reaction period as described below at room temperature, the reactions were stopped by the addition of 25 *μ*l of 100 mM EDTA, 10 *μ*g/ml Alphascreen streptavidin donor beads and 10 *μ*g/ml acceptor beads described below in 62.5 mM HEPES, pH 7.4, 250 mM NaCl, and 0.1% BSA. Plates were incubated in the dark for more than 12 h and then read by EnVision 2102 Multilabel Reader (PerkinElmer). The well containing substrate and enzyme without compound was used as total reaction control. The reaction conditions for these 10 kinases were optimized for each kinase: VEGFR2 (19 ng/ml of enzyme, 10 *μ*M ATP, 10 min reaction, PY-100 conjugated acceptor beads (PY-100)); PDGFRα (50 ng/ml of enzyme, 10 *μ*M ATP, 30 min reaction, PT66 conjugated acceptor beads (PT66)); cSrc (0.33 ng/ml of enzyme, 2 *μ*M ATP, 10 min reaction, PY-100).

**Molecular docking study**

Molecular docking of compounds **9**-**27** into the three dimensional X-ray structure of human EGFR and HER2 (PDB code: 1M17 and 3RCD, respectively) was carried out using the Discovery Studio (version 3.5) as implemented through the graphical user interface DS-CDOCKER protocol [3]. The three-dimensional structures of the aforementioned compounds were constructed using Chem. 3D ultra 12.0 software [Chemical Structure Drawing Standard; Cambridge Soft corporation, USA (2010)], then they were energetically minimized by using MMFF94 with 5000 iterations and minimum RMS gradient of 0.10. The crystal structures of two HER proteins complex were retrieved from the RCSB Protein Data Bank (http://www.rcsb.org/pdb/home/home.do). All bound waters and ligands were eliminated from the protein and the polar hydrogen was added to the proteins.

**References**

1. Saitoh M, Kunitomo J, Kimura E, Hayase Y, Kobayashi H, et al. (2009) Design, synthesis and structure-activity relationships of 1,3,4-oxadiazole derivatives as novel inhibitors of glycogen synthase kinase-3beta. Bioorg Med Chem 17: 2017-2029.

2. Domarkas J, Dudouit F, Williams C, Qiyu Q, Banerjee R, et al. (2006) The combi-targeting concept: synthesis of stable nitrosoureas designed to inhibit the epidermal growth factor receptor (EGFR). J Med Chem 49: 3544-3552.

3. Wu G, Robertson DH, Brooks CL 3rd, Vieth M (2003) Detailed analysis of grid-based molecular docking: A case study of CDOCKER-A CHARMm-based MD docking algorithm. J Comput Chem 24: 1549-1562.
